# Determination of Antioxidant, Anti-Alzheimer, Antidiabetic, Antiglaucoma and Antimicrobial Effects of Zivzik Pomegranate (*Punica granatum*)—A Chemical Profiling by LC-MS/MS

**DOI:** 10.3390/life13030735

**Published:** 2023-03-09

**Authors:** Hasan Karagecili, Ebubekir İzol, Ekrem Kirecci, İlhami Gulcin

**Affiliations:** 1 Department of Nursing, Faculty of Health Sciences, Siirt University, 56100 Siirt, Turkey; 2 Bee and Natural Products R & D and P & D Application and Research Center, Bingöl University, 12000 Bingol, Turkey; 3 Department of Chemistry, Faculty of Science, Ataturk University, 25240 Erzurum, Turkey; 4 Department of Basic Medical Sciences, Faculty of Medicine, Microbiology, Kahramanmaraş Sütçü İmam University, 46050 Kahramanmaras, Turkey

**Keywords:** Zivzik pomegranate, enzyme inhibition, *Punica granatum*, antioxidant, α-glycosidase, acetylcholinesterase, carbonic anhydrase, LC-MS/MS analysis

## Abstract

Zivzik pomegranate (*Punica granatum*) has recently sparked considerable interest due to its nutritional and antioxidant properties. To evaluate the antioxidant capacities of *P. granatum* juice, ethanol (EEZP), and water (WEZP) extracts from peel and seed, the antioxidant methods of 2,2′-azino-bis-3-ethylbenzthiazoline-6-sulphonic acid radical (ABTS^•+^) scavenging, 1,1-diphenyl-2-picrylhydrazyl free radical (DPPH^•^) scavenging, Fe^3+^-2,4,6-tris(2-pyridyl)-S-triazine (TPTZ) reducing, Fe^3+^ reducing, and Cu^2+^ reducing methods were used. The antioxidant capacities of samples were compared with the most commonly used synthetic antioxidants, i.e., BHA, BHT, α-tocopherol, and Trolox. In terms of setting an example, the IC_50_ values of EEZP for ABTS^•+^ and DPPH^•^ scavenging activities were found to be lower than standards, at 5.9 and 16.1 μg/mL, respectively. The phenolic and flavonoid contents in EEZP peel were 59.7 mg GAE/g and 88.0 mg QE/g, respectively. Inhibition of α-glycosidase, α-amylase, acetylcholinesterase, and human carbonic anhydrase II (hCA II) enzymes was also investigated. EEZP demonstrated IC_50_ values of 7.3 μg/mL against α-glycosidase, 317.7 μg/mL against α-amylase, 19.7 μg/mL against acetylcholinesterase (AChE), and 106.3 μg/mL against CA II enzymes. A total of 53 phenolic compounds were scanned, and 30 compounds were determined using LC-MS/MS. *E. coli* and *S. aureus* bacteria were resistant to all four antibiotics used as standards in hospitals.

## 1. Introduction

Pomegranate (*Punica granatum* L.) is an antiquity fruit that is primarily grown in western Asia, although it is also grown in other parts of the world, including the Mediterranean region. Its utilization has been linked to a variety of health advantages since ancient times [1,2]. Pomegranates are members of the Punicaceae family and have distinctive characteristics. Unsaturated–polyunsaturated fatty acids, vitamins, sugar, polysaccharides, polyphenols, and minerals can all be found in pomegranate seeds. Pomegranate seed oil in particular contains significant amounts of phenolic compounds, fatty acids, linoleic acid, gallic acid, and ellagic acid [3]. Pomegranate is one of the fruits that contain significant amounts of bioactive phenolic compounds, which are frequently used as botanical components in dietary supplements and herbal medicines [4]. Anthocyanins, anthocynidins, proanthocyanidins, flavonoids, vitamins, sterols, lignans, saccharides, fatty acids, organic acids, terpenes, and terpenoids are just a few of the bioactive components of pomegranates consumed in the human diet. In particular, proanthocyanidins, which have a considerable impact on human health, were investigated, focusing on their systemic lipid-lowering effects, as well as their hypoglycemic and anti-inflammatory abilities in the intestinal epithelium [5]. Other bioactive components include ellagic acid and its derivative, gallic acid. In addition to ellagic acid and its derivatives, ellagitannins and gallotannins are important bioactive components of *P. granatum* [6]. Likewise, because of their well-known potential biological and pharmaceutical properties, secondary metabolites of plants have been widely used in traditional medicine. These metabolites play protective roles in plants and exhibit various biological and pharmaceutical traits with positive effects on health [7].

Antioxidant defenses such as enzymatic antioxidants and antioxidant food ingredients are present in all aerobic organisms and are used to either remove or repair damaged molecules [8,9,10]. By delaying the lipid peroxidation process, which is one of the main causes of the degradation of pharmaceutical and food items during processing and storage, antioxidants can remove free radicals and lengthen shelf life. The effects of free radicals and ROS can be prevented by antioxidants [11]. Enzymes and antioxidant components make up the antioxidant defense system. They have the ability to replace or fix broken biomolecules in living things such as lipids, carbohydrates, nucleic acids, and proteins. The oxidation of these biomolecules is postponed, avoided, and inhibited by antioxidants. They consist of phenols and polyphenols as potent substances that lessen or neutralize the harmful and undesirable effects of ROS [12,13]. However, the human body can be assisted in reducing oxidative damage caused by free radicals and ROS by antioxidant supplements or foods [14]. As free radicals or active oxygen scavengers, many antioxidant compounds found naturally in plant sources have been identified. The search for natural antioxidants for use in food or medicine has recently attracted increasing attention because synthetic antioxidants, the use of which is restricted due to their side effects such as carcinogenicity, are becoming harder to find [15]. Recently, propyl gallate, *tert*-butyl hydroquinone, butylated hydroxyanisole (BHA), and butylated hydroxytoluene (BHT) have become the most commonly used antioxidants. However, BHA and BHT have been subject to legislative restrictions because of concerns about their carcinogenic and toxic effects [16]. As a result, there is an increase in consumer preference for natural antioxidants, as well as an increased interest in natural and safer antioxidants for food applications, both of which have sparked efforts to investigate natural sources of antioxidants [17,18]. The leaves, roots, seeds, and fruits of most plants contain natural antioxidants. Numerous fruits and vegetables are rich in substances such as polyphenols, ascorbic acid, carotenoids, and tocopherols that have positive effects on health. Consuming fruits and vegetables can lower the risk of developing chronic diseases such as cardiovascular disease and cancer [19]. Medicinal plants, which have been the subject of many studies to date, are among the most significant natural antioxidant sources. Medicinal plants contain a high number of phenols. Cereals, plants, and fruits are the main sources of natural antioxidants in the human diet [20]. Phenolic compounds are plant secondary metabolites that prevent degenerative disorders such as cataracts, cardiovascular disease, cancer, hypercholesterolemia, rheumatoid arthritis, diabetes, and arteriosclerosis [21,22].

Alzheimer’s disease (AD) is regarded as one of the most pressing global health concerns of our time. Numerous AD treatment strategies have been developed. The inhibition of acetylcholinesterase (AChE) and butyrylcholinesterase (BChE) is one of the most important strategies [23,24]. In particular, in several medical conditions, such as carcinogenesis, coronary atherosclerosis, AD, and age-related disorders, lipid peroxidative destruction has been linked to biologically active substances with antioxidant effects. AChE and BChE are activity parameters that are still considered to be a part of prophylaxis to treat neurological disorders associated with AD [25]. Alzheimer’s disease (AD) is initially distinguished by the development of memory loss and other cognitive disorders and is believed to be connected to acetylcholine (ACh) deficiency, inflammation, and oxidative stress. Consuming plants with antioxidant capabilities can therefore stop the progression of AD and neurodegeneration [26].

It is thought that these disorders can be cured by inhibiting the essential enzymes linked to them. However, the synthetic drugs that are used to inhibit these important enzymes have a lot of negative side effects [27]. Researchers were encouraged to find an alternative natural product with fewer or no negative effects in an effort to solve this issue [28]. Natural substances such as AChE inhibitors (AChEIs) have frequently been used in clinical trials, particularly for the treatment of AD. Phenolic substances served as the first drugs for the treatment of AD and were also discovered to be AChEIs [29].

Inhibiting enzymes that hydrolyze carbohydrates, such as α-glucosidase and α-amylase, is one of the current methods for treating T2DM. By delaying glucose absorption, postprandial plasma glucose levels can be lowered, and hyperglycemia can be suppressed [30]. The hydrolysis of both oligosaccharides and polysaccharides into monosaccharide components such as glucose and fructose is accomplished by the enzyme glycosidase, which is released from intestine cells. As a result, the control of type 2 diabetes mellitus and hyperglycemia requires the use of α-glycosidase inhibitors (α-GIs) [31].

Carbonic anhydrase enzymes (CAs) are Zn^2+^-including metalloenzymes, which catalyze the reversible hydration of carbon dioxide (CO_2_) to proton and bicarbonate (HCO_3_^−^) [32]. Numerous biochemical functions such as ureagenesis, lipogenesis, and gluconeogenesis are carried out by CAs [33]. CA inhibition has therapeutic uses in the treatment of infection, convulsions, glaucoma, and cancer [34]. They also maintain fluid balance throughout the body, especially in the eyes, kidneys, and stomach. High intraocular pressure (IOP) due to glaucoma (IOP) can be relieved with the use of inhibitors of carbonic anhydrase (CAIs) [35].

With the current study, we sought to investigate the chemical components and biological activities of *P. granatum* obtained from the Zivzik village of Siirt province in the southeastern Anatolian region of Turkey. The following steps were taken to accomplish this: (a) The phenolic and flavonoid profiles of *P. granatum* were determined using LC-MS/MS analysis; (b) the antioxidant abilities of *P. granatum* were measured using DPPH, DMPD, ABTS, Cu^2+^ reducing (CUPRAC), Fe^3+^ reducing, Fe^3+^-TPTZ reducing (FRAP), and Folin–Ciocalteu techniques; (c) the inhibitory effect of *P. granatum* on some metabolic enzymes, including AChE, hCA II, and α-glycosidase, was investigated for determination of possible relationships with AD, glaucoma, and diabetes mellitus; and (d) Gram-positive (*Staphylococcus aureus*) and Gram-negative (*Escherichia coli*) microorganisms were used to test the antimicrobial activity.

## 2. Materials and Methods

### 2.1. Chemicals

Acetylcholinesterase, acetylcholine iodide, α-glycosidase, p-nitrophenyl-D-glycopyranoside, DPPH (1,1-diphenyl-2-picryl-hydrazyl), ABTS (2,2-azino-bis 3-ethylbenzthiazoline-6-sulfonic acid), neocuproine (2,9-dimethyl-1,10-phenanthroline), BHT (butylated hydroxytoluene), BHA (butylated hydroxyanisole), α-tocopherol, Trolox, (Ferrozine) 3-(2-pyridyl)-5,6-bis(4-phenyl-sulfonic acid)-1,2,4-triazine, (TCA) trichloroacetic acid, and standard phenolic compounds for LC-MS/MS were purchased from Sigma (Sigma-Aldrich GmbH, Steinheim, Germany). The other materials were procured from Sigma-Aldrich or Merck.

### 2.2. Plant Materials

Zivzik pomegranate (*Punica granatum*) was defined as the Siirt ecotype by Assoc. Prof. Dr. Mehmet Fidan from the Siirt University Department of Biology. Zivzik pomegranates were obtained from Dişlipinar village (Zivzik) in the Şirvan district of Siirt province (altitude: 764 m (2506 ft)). *P. granatum* ethanol and water extract were dissolved in ethanol to determine their antioxidant activities and in DMSO for tests of enzyme inhibition due to the potential inhibitory effects of ethanol.

### 2.3. Preparation of Zivzik Pomegranate (Punica granatum)’s Extracts 

The extraction procedure was performed as previously described [36]. Water extracts of *P. granatum* (WEZP) were prepared using 100 mL of distilled water and 25 g of dried *P. granatum* peel and seeds that had been ground in a mill. This mixture was boiled for 20 min in a magnetic stirrer. The filtrates of the extracts were frozen and lyophilized in a lyophilizator at −50 °C under a pressure of 5 mmHg (Labconco, Freezone).

For ethanol extracts of *P. granatum* (WEZP), 25 g of dried *P. granatum* peel and seeds were milled before being combined with 100 mL of ethanol and stirred in a magnetic stirrer for 1 h. Filtrates were collected after the extracts had been filtered. A rotary evaporator (RE 100 Bibby, Stone Staffordshire, England) operating at 50 °C was used to remove the ethanol. Before being used in experimental studies, all of the extracts were kept in a dark plastic bottle at a temperature of 20 °C [37]. The yield of *P. granatum* extraction was calculated using the following equation:

Yield = Weight of *P. granatum* extract (g)/weight of raw extract (g) ×100%

The yield of *P. granatum* extracts were calculated as follows: WEZP peel = 9.4/15 × 100 = 62.7%; WEZP seed = 8.6/15 × 100 = 57.3%; EEZP peel = 4/15 × 100 = 26.7%; EEZP seed = 2.92/15 × 100 = 19.5%. In order to obtain *P. granatum* juice, first, *P. granatum* were peeled, and pomegranate seeds were obtained. Then, *P. granatum* juice was obtained by pressing the *P. granatum* arils through a cheesecloth.

### 2.4. Total Phenolic Contents

**Figure 1:**
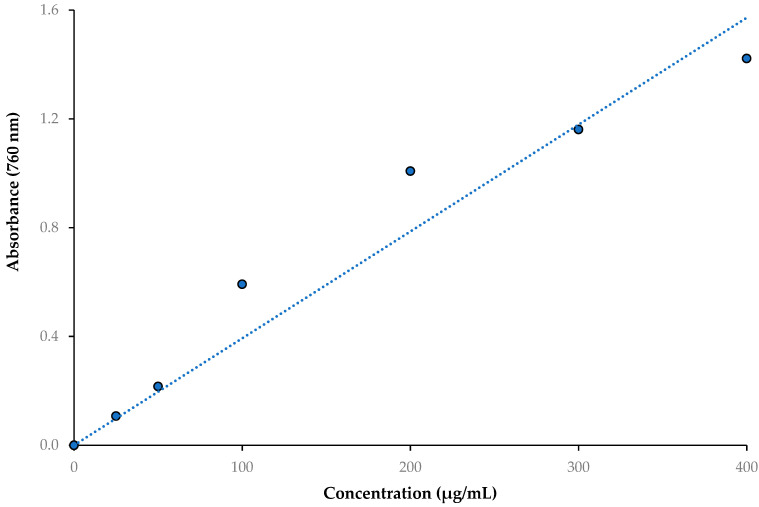
The standard curve of gallic acid for total phenolic contents (r^2^: 0.9408).

The method described by Singleton and Rossi [38] was used to quantify the phenolics in the WEZP and EEZP peel and seed and *P. granatum* juice with a few minor modifications [39,40]. First 0.5 mL of each extracted sample was transferred to Folin–Ciocalteu reagent (FCR, 1.0 mL). The solution was then thoroughly blended and neutralized with carbonate (0.5 mL, 1%). After two hours of incubation in the dark at room temperature, the absorbances were measured at 760 nm in comparison to a blank sample, which included water. The phenolic content was expressed as milligrams of gallic acid equivalents (GAE) per gram of WEZP, EEZP, and *P. granatum* juice. The standard curve of gallic acid for total phenolic contents (r^2^: 0.9408) is presented in Figure 1.

### 2.5. Total Flavonoid Contents

**Figure 2:**
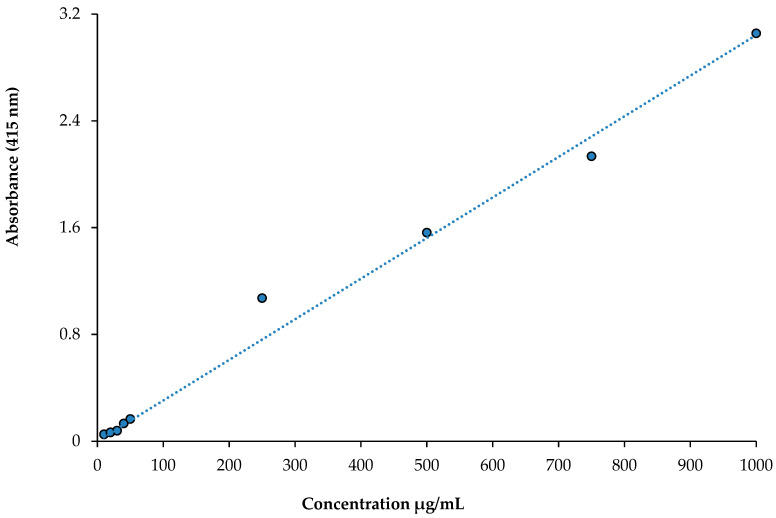
The standard curve of quercetin for total flavonoid contents (r^2^: 0.9877).

A class of polyphenolic substances known as flavonoids is widely distributed in plants and frequently found in the human diet. Based on a previously described method [41], a colorimetric assay was used to estimate the total flavonoid contents in WEZP, EEZP, and *P. granatum* juice. To this end, 0.5 mL of sample was combined with 1.5 mL of 95% methanol. Then, 0.5 mL CH_3_COOK (1.0 M) and 2.3 mL of deionized water were combined with 1.5 mL of 10% Al(NO_3_), and the samples were vortexed. Then, the vortexed samples were kept at 25 °C for 40 min in the dark. Absorbance measurements were taken at a wavelength of 415 nm. Quercetin equivalents (QE) are reported as mg per gram of extract in this study. The standard curve of total flavonoid contents is obtained from Figure 2.

### 2.6. Analaysis of Polyphenolic Composition by LC-MS/MS

#### 2.6.1. Sample Preparation

First, 100 mg of each WEZP and EEZP was dissolved in 5 mL of water–ethanol (50:50 *v*/*v*) in a volumetric flask, and 1 mL of this solution was added to another volumetric flask with a capacity of 5 mL. Then, 100 μL of *P. granatum* extracts were added and diluted to the volume with water–ethanol (50:50 *v*/*v*). An aliquot of 1.5 mL from the final solution was transferred into a vial with a cap, and 10 μL of the sample was injected into the LC-MS/MS. Throughout the experiment, the samples in the autosampler were kept at 15 °C [42].

#### 2.6.2. Method Validation Parameters and LC-MS/MS Analysis

The analytical approach utilized in this investigation was in accordance with the latest studies. The LC-MS/MS study was carried out by the Dicle University Central Research Laboratory. This chromatographic method was successfully carried out by Yılmaz [43] and adapted for *P. granatum* ethanol extracts. A total of 53 phytochemical standards were obtained as reference from Sigma-Aldrich (Steinheim, Germany). They were used to analyze phytochemicals in EEZP and WEZP.

### 2.7. Fe^3+^ Reducing Capacity

The Fe^3+^ reducing capacities of *P. granatum,* EEZP, WEZP, and *P. granatum* juice were assessed on the basis of the method proposed by Oyaizu [44], as also previously described in [45]. In a summary, various concentrations of samples in 0.75 mL of distilled water (10–30 μg/mL) were added into the same volume of buffer solution (1.25 mL, pH 6.6; 0.2 M) and 1.25 mL of K_3_Fe(CN)_6_ (1%, *w*/*w*). Trichloroacetic acid (TCA) (1.25 mL, 10%) was used to acidify the mixture after it has been incubated at 50 °C for 30 min. The absorbances of the fruit extracts were recorded at 700 nm after an aliquot of 0.1%, 0.25 mL, and FeCl_3_ solution had been added to the mixture. Phosphate buffer solution was used as a blank sample. Activity measurements for the Fe^3+^ reducing ability at each concentration were conducted in triplicate.

### 2.8. Cu^2+^ Reducing Capacity

The Cu^2+^ reducing abilities of EEZP, WEZP, and *P. granatum* juice were measured according to the method used by Apak et al. [46], which was thoroughly described in [47], To this end, the same volumes of 0.25 mL of CuCl_2_ solution (10 mM), 0.25 mL of neocuproine solution (7.5 mM), and 0.25 mL of acetate buffer (1.0 M) were added to the EEZP and WEZP solutions (10–30 μg/mL) in a test tube. The total volumes of mixtures were adjusted to 2 mL with distilled water and vigorously mixed. Then, the glass tubes were closed and retained at 25 °C until use in experiments. Finally, after 30 min, the absorbances were spectrophotometrically recorded at 450 nm. Acetate buffer solution was used as a blank sample. Increased reaction mixture absorbance suggests increased reduction capacity. Activity measurements for Cu^2+^ reducing ability at each concentration conducted in triplicate [48].

### 2.9. Fe^3+^-TPTZ Reducing Capacity

The Fe^3+^-TPTZ reducing capacity of EEZP, WEZP, and *P. granatum* juice in acidic solution were measured at 593 nm [49]. TPTZ solution (10 mM, 2.25 mL) with FeCl_3_ (20 mM, 2.25 mL) in acetate buffer made up the FRAP reagent solution (2.5 mL, pH 3.6, 0.3 M). The mixture was then incubated at 37 °C in the dark for 30 min after EEZP, WEZP, and *P. granatum* juice (10–30 g/mL) were dissolved in buffer solution (5 mL). The absorbance of the samples was then measured. Activity measurements for Fe^3+^-TPTZ reducing ability at each concentration were conducted in triplicate [50].

### 2.10. DPPH^•^ Scavenging Activity

The bleaching of a purple DPPH solution in methanol allows for the presence of certain pure antioxidant compounds with hydrogen-atom- or electron-donating properties to be determined. Stable DPPH^•^ is the reagent used in this spectrophotometric assay [51]. The method described by Blois [52], as previously applied by Gulcin [53], was used with minor modification to estimate the DPPH^•^ free radical scavenging capacity of EEZP, WEZP, and *P. granatum* juice; a stable free radical called DPPH was monitored for bleaching at a specific wavelength while the sample was present. The DPPH^•^ solution was prepared daily. Aluminum foil was used to cover the solution flask, which was stirred for 16 h at 4 °C while being kept in the dark. Shortly after preparing a 0.1 mM DPPH^•^ solution in ethanol, 0.5 mL of this solution was combined with 2 mL of EEZP, WEZP, and *P. granatum* juice at various concentrations (10–30 g/mL). After being vortexed, the samples were incubated at 30 °C in the dark for 30 min. Absorbance was measured at 517 nm in comparison to blank samples. The scavenging of DPPH free radicals is indicated by a decrease in absorbance [54]. When DPPH is reduced by an antioxidant or another radical species, its absorption falls below that of the radical form, which absorbs at 517 nm. The absorbance at 517 nm decreased proportionately to an increase in DPPH’s non-radical forms when a hydrogen atom or electron was transferred to the odd electron [55]. Absorbance decreases indicate that DPPH is actively scavenging free radicals. Activity measurements for DPPH radical scavenging activity at each concentration were conducted in triplicate [56].

### 2.11. ABTS^•+^ Scavenging Activity

A relatively stable free radical, ABTS, also decolorizes in its non-radical state. The method of Re et al. [57] was used to determine ABTS^•+^ scavenging activity. This technique involves adding an antioxidant to a prepared ABTS radical solution, and after a set amount of time, the remaining ABTS^•+^ is measured spectrophotometrically at 734 nm [58]. Then, 2 mM ABTS in water was combined with 2.45 mM potassium persulfate (K_2_S_2_O_8_) to create ABTS^•+^, which was then left to sit for 6 h at room temperature in the dark. The ABTS started to oxidize right away, but it took over 6 h for the absorbance to reach its peak and stabilize. Under storage conditions at room temperature in the dark, the radical cation is stable in this form for longer than two days. In order to perform the assay, the solution was diluted in phosphate buffer (pH 7.4), providing an absorbance of 0.700 ± 0.025 at 734 nm and equilibrated to 30 °C, the temperature at which all assays were carried out. Then, 3 mL of EEZP, WEZP, and *P. granatum* juice in ethanol at 10–30 μg/mL were combined with 1 mL of the ABTS^•+^ solution. After mixing for 30 min, the absorbance was measured, and the radical scavenging percentage was computed for each concentration in comparison to a blank containing no scavenger. The percentage reduction in absorbance was used to determine the degree of decolorization. Activity measurements for ABTS radical scavenging activity at each concentration were conducted in triplicate [59].

### 2.12. Enzyme Inhibition Studies

#### 2.12.1. Acetylcholinesterase Inhibition Study

The cholinergic enzyme-inhibitory abilities of EEZP, WEZP, and *P. granatum* juice were determined using Ellman’s methodology [60] as described in a previous study [61]. This was accomplished using AChE serum from electric eels. Briefly, a specific *P. granatum* concentration (10–30 μg/mL) in buffer (1.0 M Tris/HCl, 100 μL, pH 8.0) was transferred to the enzyme solution (50 μL, 5.32 10^−3^ EU). The mixtures were kept at 20 °C for 10 min. Then, 50 μL of mixtures containing DTNB (5,5′-dithio-bis(2-nitro-benzoic acid) (0.5 mM)) and acetylthiocholine iodide (AChI) was added. The reaction medium was then started, and the mixture’s absorbances were measured spectrophotometrically at 412 nm [62].

#### 2.12.2. α-Glycosidase Inhibition Study

The inhibitory abilities of the WEZP, EEZP, and *P. granatum* juice on α-glycosidase were determined based on the method of Tao et al. [63], as described in detail in [64]. Various amounts of WEZP, EEZP, and *P. granatum* juice were transferred to phosphate buffer (75 μL, pH 7.4) for this purpose. Then, 20 μL of α-glycosidase solution was transferred to the same buffer and incubated for 10 min. The final mixture was mixed with 50 μL of p-nitrophenyl-D-glycopyranoside (p-NPG) dissolved in the same buffer. The mixture was then incubated again at room temperature (37 °C), and the absorbances were measured at 405 nm against a blank sample made up of phosphate buffer.

#### 2.12.3. α-Amylase Inhibition Study

The inhibitory effects of WEZP, EEZP, and *P. granatum* juice on α-amylase were measured according to Xiao et al [65]. Briefly, 40 mL of 0.4 M alkaline solution was used to dissolve 1 g of starch, which was then heated for 30 min at 80 °C. The pH of the mixture was adjusted to 6.9, and the total volume was adjusted to 100 mL with deionized water. Then, different amounts of WEZP, EEZP, and *P. granatum* juice and 35 μL of starch prepared in buffer solution (pH 6.9) were mixed. Then, 20 μL of enzyme was added to the mixture and incubated at 40 °C for 60 min. Finally, 50 μL of HCl (0.1 M) was added to the mixture, and the reaction was stopped. The absorbance of samples was measured at 580 nm. The blank sample contained buffer solution (pH 6.9).

#### 2.12.4. hCA Inhibition Study

The sepharose-4B-L-tyrosine sulfanilamide affinity technique was used to separate and purify CA II isoenzymes using human blood samples, as previously reported [66]. The protein levels were determined at 595 nm using the Bradford method after the enzymes had been purified [67]. The spectrophotometric Verpoorte’s method (Shimadzu, UVmini-1240 UV–VIS) was used to perform CA activity [68]. Acetazolamide (AZA) was utilized as a reference standard [69].

### 2.13. Antimicrobial Studies

#### 2.13.1. Microorganisms to Be Used in the Study

Microorganisms that can be potentially harmful to humans were used in this study. Gram-positive bacteria (*S. aureus* ATCC 25923) and Gram-negative bacteria (*E. coli* clinical isolate) were used for the assessment of antibacterial activity [70]. Bacterial strains were derived from stock cultures (clinical isolates and standard strains) of Kahramanmaras Sutcu İmam University Faculty of Medicine, Department of Medical Microbiology, Microbiology Laboratory.

#### 2.13.2. Identification of *E. coli* Clinical Isolates

The identification of *E. coli* clinical isolates was realized according to the method of Deniz et al. [71]. Pathogen bacterial isolations from various clinical samples collected from patients and delivered to the laboratory under sterile conditions were inoculated on blood agar and EMB agar, and the media were incubated at 37 °C for 48 h. Colonies of *E. coli* bacteria grown in culture media were identified as species by Gram staining, biochemical tests, and the BD Phoenix 100 identification system.

#### 2.13.3. Antimicrobial Activity Determination

The antimicrobial activity of the WEZP, EEZP, and *P. granatum* juice was determined by a disk diffusion method [72]. The test microorganism agar cultures were prepared in accordance with the procedure described by Gulcin et al. [73]. Bacterial strains were grown on blood agar medium (Oxoid CM55, Basingstoke, Hampshire, UK). In the study, pathogens to be evaluated were inoculated into Tryptone soy broth (Oxoid CM129, Basingstoke, Hampshire, UK). Facultative anaerobes and aerobes, including some fungi, were cultivated using tryptone soy broth, a highly nutritive and versatile medium that is recommended for general laboratory use. Prepared cultures were incubated for 24 h at 37 °C. For the antimicrobial test, 50 μL of WEZP, EEZP, and *P. granatum* juice was added to sterile 6 mm diameter filter paper discs, and susceptibility measurements were conducted on Mueller Hinton agar (Oxoid CM337, Basingstoke, Hampshire, UK) medium with the diffusion technique prescribed in Clinical and Laboratory Standards (CLSI 2018).

The growth inhibition zones around the discs containing antibiotics and WEZP, EEZP, and *P. granatum* juice were measured and recorded. The presence of antimicrobial activity was shown by clear zones of inhibition surrounding the discs [74]. Plant extracts, amoxicillin-clavulanic acid (20/10 μg/disc), gentamicin (10 μg/disc), ampicillin-sulbactam (10/10 μg/disc), and ciprofloxacin (5 μg/disc, BD BBL™ Sensi-Disc™) were compared with standard antimicrobial discs. Antimicrobial test results were analyzed according to the references suggested by the Clinical and Laboratory Standards [75].

### 2.14. Statistical Analysis

All experiments are repeated three times for each sample. The results are reported as the mean ± SD. (n = 3) and were evaluated using one-way ANOVA followed by Tukey’s post hoc test; *p* < 0.05 was considered statistically significant.

## 3. Results

### 3.1. Total Phenolics, Total Flavonoids, and LC-MS/MS Analysis Results

The phenolic and flavonoid contents in EEZP peel were measured as 59.7 mg GAE/g and 88.0 mg QE/g, respectively, in this study. Between 6.36 and 1.78 mg GAE/100 mL of total phenolics were present in five different pomegranate cultivars. The total flavonoid content varied from 4.93 to 2.24 mg GAE/100 mL [2]. “Wonderful” pomegranate fruit mineral concentration, bioactivity, and internal quality were improved using foliar nutrient applications. Total phenolic content in *P. granatum* juice ranged from 2091 to 3735 mg/L GAE [76]. The polyphenol and flavonoid contents of pomegranate peel acetone extract (338 ± 20 mg/g GAE and 60.8 ± 9.3 mg/g QE, respectively) were significantly (*p* < 0.05) higher than those of water and ethanol extracts. Additionally, it was discovered that the polyphenol and flavonoid levels of acetone extract were higher than those found in methanol, ethanol, and ethyl acetate extracts of the fruit peels of various Pakistani pomegranate varieties, including “Desi”, “Kandhari”, and “Badana” [77]. In this study, *P. granatum* extracts were shown to have comparable effective amounts of polyphenolics.

**Figure 3:**
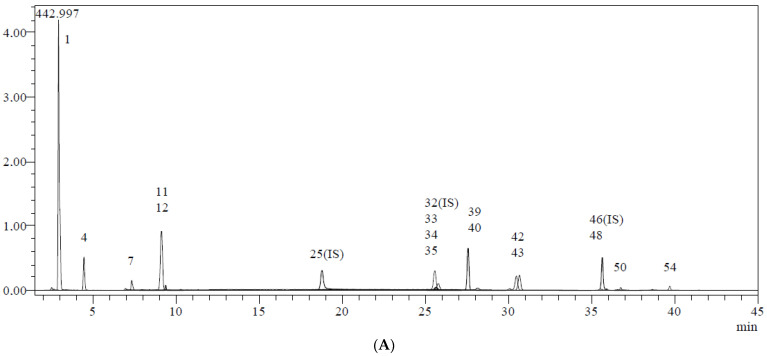

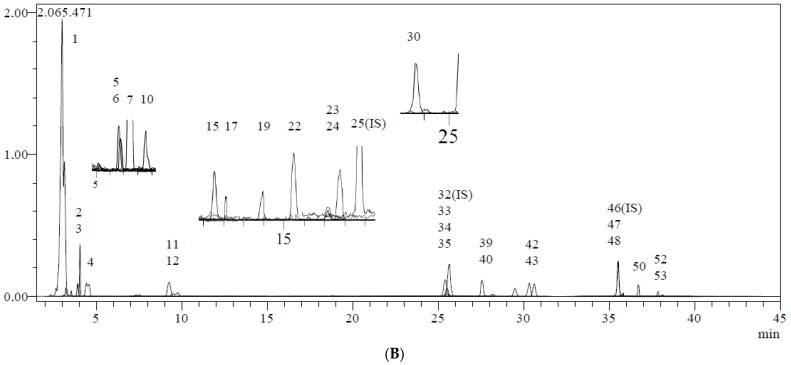
(**A**) Chromatogram of *P. granatum* water extract compounds. (**B**) Chromatogram of *P. granatum* ethanol extract compounds (1. quinic acid, 2. fumaric acid, 3. aconitic acid, 4. gallic acid, 5. epigallocatechin 7. catechin, 11. tannic acid, 12. epigallocatechin gallate, 15. epicatechin, 22. epicatechin gallate, 33. rutin, 34. isoquercitrin, 35. hesperidin, 39. ellagic acid, 40. cosmosiin, 42. astragalin, 43. nicotiflorin, 50. luteolin).

**Figure 4:**
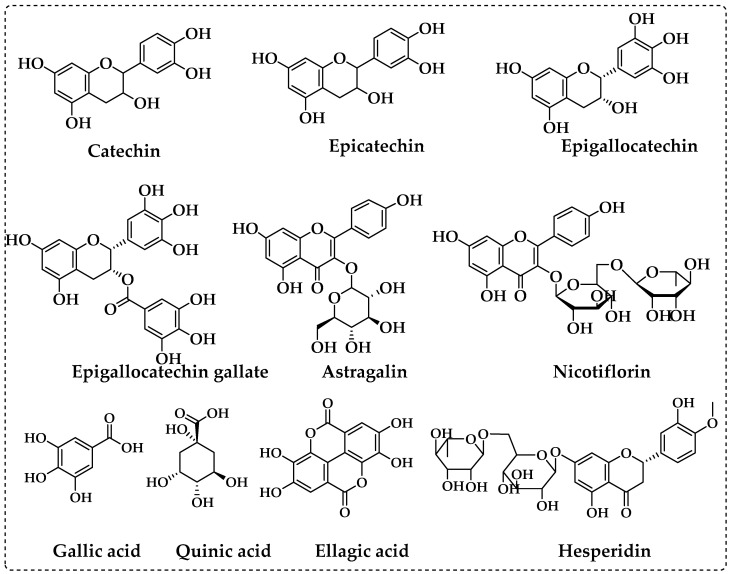
The structures of the ten most abundant phenolic compounds in *P. granatum*.

Using fifty-three phenolics as standard compounds, the LC-MS/MS method was utilized to identify the major phenolic components in *P. granatum* extracts. The elucidation of phenolic compounds was accomplished by comparison of their chromatographic behavior, UV spectra, and MS information with references, and thirty compounds were measured (Table 1 and Figure 3). Table 1 shows the mean values of each chemical based on the LC-MS/MS tests. The major compounds detected in ethanol extract of *P. granatum* were ellagic acid (199.967 mg/g), catechin (27.664 mg/g), epigallocatechin gallate (25.600 mg/g), epicatechin (24.210 mg/g), nicotiflorin (23.535 mg/g), astragalin (20.551 mg/g), gallic acid (20.021 mg/g), epigallocatechin (19.148 mg/g), quinic acid (17.460 mg/g), tannic acid (12.300 mg/g), aconitic acid (8.190 mg/g), hesperidin (6.136 mg/g), isoquercitrin (4.056 mg/g), rutin (2.732 mg/g), fumaric acid (2.128 mg/g), cosmosiin (2.036 mg/g), luteolin (1.126 mg/g), and epicatechin gallate (1.060 mg/g). Also, the compounds include protocatechuic acid, protocatechuic aldehyde, caffeic acid, vanillin, piceid, p-cumaric acid, cynaroside, quercetin, naringenin, kaempferol, apigenin, amentoflavone, gentisic acid, and chlorogenic acid were detected. Only quinic acid was in found a higher amount (44.662 mg/g) in water extract of *P. granatum*, whereas 1,5-dicaffeoylquinic acid, 4-OH-benzoic acid, vanillic acid, syringic acid, daidzin, syringic aldehyde, ferulic acid-D3-IS, ferulic acid, coumarin, sinapic acid, salicylic acid, miquelianin rutin D3-IS, *O*-coumaric acid, rosmarinic acid, genistin, quercitrin, fisetin, daidzein, quercetin-D3-IS, hesperetin, genistein, chrysin, and acacetin were not recorded in EEZP and WEZP. The chemical structures of the most plentiful phenolics in *P. granatum* are presented in Figure 4.

### 3.2. Reducing Ability Results

**Figure 5:**
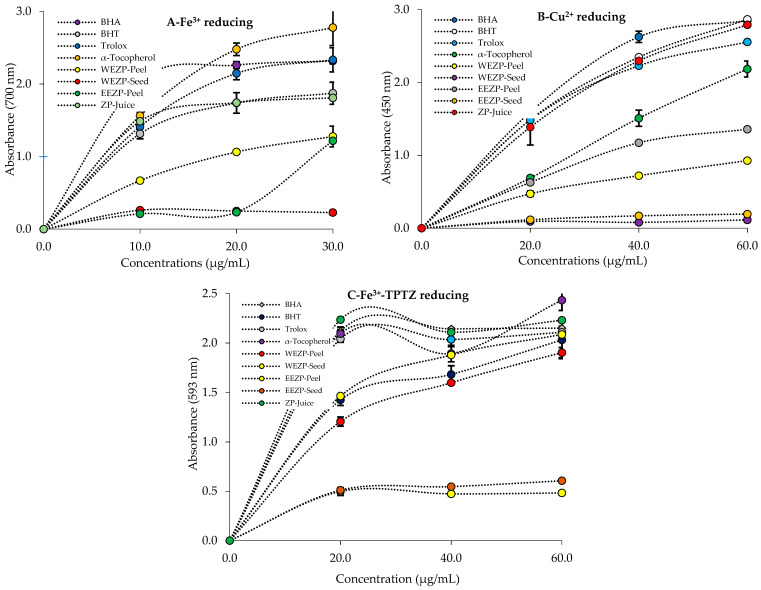
Fe^3+^ (**A**), Cu^2+^ (**B**), and Fe^3+^-TPTZ (**C**) reducing abilities of *P. granatum* and standards.

As summarized in Table 2 and Figure 5A, *P. granatum* extracts showed a potent Fe^3+^ reducing profile. However, the Fe^3+^ reducing ability of a 30 μg/mL concentration of *P. granatum* extracts, phenolic compounds, and standards decreased in the following order: α-tocopherol (2.778 ± 0.248, r^2^: 0.9999) > Trolox (2.334 ± 0.167, r^2^: 0.9997) > BHA (2.319 ± 0.041, r^2^: 0.9629) > BHT (1.873 ± 0.152, r^2^: 0.9918) > *P. granatum* juice (1.810 ± 0.149, r^2^: 0.7433) > WEZP peel (1.278 ± 0.143, r^2^: 0.9995) > EEZP peel (1.219 ± 0.028, r^2^: 0.9253) > EEZP seed (0.258 ± 0.005, r^2^:0.9712) > WEZP seed (0.229 ± 0.033, r^2^: 0.9252). All analyses were carried out in triplicate. Depending on the reducing antioxidant capacity of *P. granatum* extracts, the test solution’s yellow color in this assay shifted to various shades of green and blue.

Cu^2+^ reducing abilities of the phenolic composition in *P. granatum* extracts and juice are presented in Table 2 and Figure 5B. It was determined that there was a strong relationship between the Cu^2+^ reducing impact and different concentrations of phenolics in *P. granatum* extracts. However, at a concentration of 30 µg/mL, the significant absorbance of reducing ability was demonstrated by phenolics in *P. granatum* extracts. On the other hand, the Cu^2+^ reducing abilities of *P. granatum* extracts and standards were found as follows: BHT (2.865 ± 0.038, r^2^: 0.9991) > BHA (2.849 ± 0.020, r^2^: 0.9999) > *P. granatum* juice (2.790 ± 0.045, r^2^: 0.9999) > Trolox (2.555 ± 0.022, r^2^: 0.9987) > α-tocopherol (2.185 ± 0.110, r^2^: 0.9986) > WEZP peel (0.927 ± 0.022, r^2^: 0.9965) > EEZP peel (0.878 ± 0.017, r^2^: 0.9967) > EEZP seed (0.194 ± 0.008, r^2^: 0.9974) > WEZP seed (0.114 ± 0.034, r^2^: 0.8485).

An Fe^3+^-TPTZ reducing assay was used to determine the reducing abilities of *P. granatum* extracts and standards. The reduction powers of samples dropped in the following sequence according to the results provided in Table 2 and Figure 5C: α-tocopherol (2.434 ± 0.103, r^2^: 0.8714) > ZP juice (2.230 ± 0.010, r^2^: 0.9056) > BHA (2.151 ± 0.020, r^2^: 0.9367) > Trolox (2.108 ± 0.026, r^2^: 0.9291) > EEZP peel (2.086 ± 0.080, r^2^: 0.9866) > BHT (2.031 ± 0.190, r^2^: 0.9670) > WEZP peel (1.903 ± 0.052, r^2^: 0.9875) > EEZP seed (0.606 ± 0.011, r^2^: 0.9471) > WEZP seed (0.483 ± 0.023, r^2^: 0.9124). In this method, the higher the absorbance readings, the better the test samples’ ability to reduce.

### 3.3. Radical Scavenging Results

**Figure 6:**
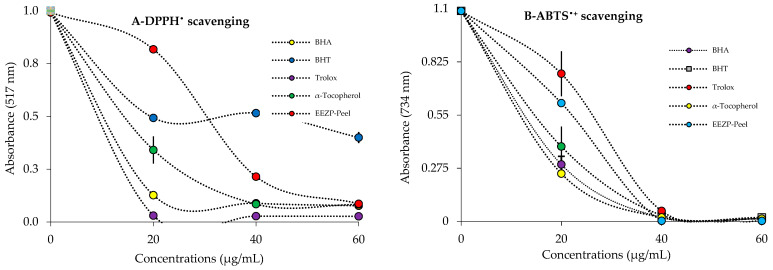
Radical scavenging effects of *P. granatum* and positive controls. (**A**) DPPH^•^ scavenging ability; (**B**) ABTS^•+^ scavenging ability.

*P. granatum* extracts and juice are thought to have natural antioxidant potential if they have DPPH^•^ scavenging ability. The DPPH^•^ scavenging activity of *P. granatum* extracts was measured, and the IC_50_ value was derived (Table 3, Figure 6A). *P. granatum* extracts and juice demonstrated concentration-dependent radical scavenging activity (Figure 6A). The DPPH^•^ scavenging capability of *P. granatum* extracts, juice, and standards was decreased as follows: ascorbic acid (IC_50_: 5.82 μg/mL) > Trolox (IC_50_: 6.03 μg/mL) > BHA (IC_50_: 6.86 μg/mL) > α-Tocopherol (7.70 μg/mL) > EEZP peel (IC_50_: 16.10 μg/mL) WEZP peel (IC_50_: 31.50 μg/mL) > BHT (IC_50_: 49.50 μg/mL). DPPH^•^ scavenging abilities of WEZP seed, EEZP seed, and *P. granatum* juice could not be measured due to the color blur that occurred during measurements.

IC_50_ values of ABTS^•+^ scavenging of *P. granatum* extracts and juice and reference radical scavenger agents such as Trolox, α-tocopherol, BHT, and BHA were detected in the following range: EEZP peel (IC_50_: 5.90 μg/mL, r^2^: 0.9669) > BHA (IC_50_: 6.35 μg/mL, r^2^: 0.9746) > WEZP peel (IC_50_: 8.80 μg/mL, r^2^: 0.9178) > ascorbic acid (IC_50_: 11.74 μg/mL, r^2^: 0.9983) > BHT (IC_50_: 12.60 μg/mL, r^2^: 0.9995) Trolox (IC_50_:1 6.50 μg/mL, r^2^: 0.9775) > α-Tocopherol (18.72 μg/mL, r^2^: 0.9347) (Table 3 and Figure 6B). As with DPPH^•^ scavenging measurements, ABTS^•+^ scavenging abilities of WEZP seed, EEZP seed, and *P. granatum* juice could not be measured due to the color blur that occurred during measurements.

### 3.4. Enzyme Inhibition Results

For α-glycosidase enzyme, *P. granatum* extracts and juice exhibited effective inhibition effects. From this perspective, EEZP peel demonstrated an IC_50_ value of 7.3 μg/mL (r^2^: 0.9941), WEZP seed exhibited an IC_50_ value of 7.3 μg/mL (r^2^: 0.8819), *P. granatum* juice displayed an IC_50_ value of 27.1 μg/mL (r^2^: 0.9665), and WEZP peel showed an IC_50_ value of 28.8 (r^2^: 0.9420) (Table 4). However, the IC_50_ value could not be determined for EEZP seed. On the other hand, acarbose, as a standard for α-glycosidase and α-amylase, showed a value of 22,800 μM against α-glycosidase [63].

In inhibition studies conducted using similar methods, *P. granatum* extracts and juice were assayed for α-amylase inhibition ability, the results of which are presented in Table 4. For α-amylase enzyme, EEZP peel demonstrated an IC_50_ value of 317.7 μg/mL (r^2^: 0.7778), WEZP seed exhibited an IC_50_ value of 375.8 μg/mL (r^2^: 0.8193), WEZP peel showed an IC_50_ value of 494.3 μg/mL (r^2^: 0.7705), and *P. granatum* juice displayed an IC_50_ value of 70.1 μg/mL (r^2^: 0.9999) (Table 4).

In addition, dominant cytosolic CA II isoform is frequently linked to a number of illnesses, including osteoporosis, glaucoma, and renal tubular acidosis. The CA inhibitory effects of *P. granatum* extracts and juice were decreased in the following order (Table 4): WEZP seed (IC_50_: 144.5 μg/mL; r^2^: 0.9906) > EEZP peel (IC_50_: 106.3 μg/mL; r^2^: 0.9941) > *P. granatum* juice (IC_50_: 94.0 μg/mL; r^2^: 0.9909) > WEZP peel (IC_50_: 36.4 μg/mL; r^2^: 0.9957) > EEZP seed (IC_50_: 30.4 μg/mL; r^2^: 0.9999) > acetazolamide (IC_50_: 8.4 μg/mL; r^2^: 0.9825). AZA was employed as a control for the inhibition of CA isoenzymes [78].

AChE was the first FDA-approved therapeutic target for the AD treatment, and many drugs are currently produced and marketed for this purpose. The AChE-inhibitory capacity of *P. granatum* extracts and juice was enhanced in the following order (Table 4): *P. granatum* juice (IC_50_, 22.6 μg/mL; r^2^: 0.9951) > WEZP seed (IC_50_: 20.4 μg/mL; r^2^: 0.9851) > WEZP peel (IC_50_: 20.0 μg/mL; r^2^: 0.9976) > EEZP peel (IC_50_: 19.7 μg/mL; r^2^: 0.9869) > EEZP seed (IC_50_: 17.8 μg/mL; r^2^: 0.9976) > tacrine (IC_50_: 5.97 μg/mL; r^2^: 0.9706; as a positive control for the inhibition of cholinergic enzymes) [79].

Urinary tract infections, respiratory pneumonia, surgical site infections, bacteremia, gastrointestinal disorders, and skin infections are among the most common nosocomial infections. *Staphylococcus aureus*, as a Gram-positive microorganism, and *E. coli*, as a Gram-negative microorganism, are the most prevalent pathogens that cause these infections according to the Center for Disease Control and Prevention (Atlanta, USA) [80]. We chose to test the effectiveness of *P. granatum* extracts and juice against these microorganisms since they are notoriously difficult to eradicate due to their resistance to most antimicrobial agents. Antimicrobial results are shown in Table 5.

## 4. Discussion

A vital and important component of the human diet is phenolic chemicals, which are present in all plants. Their biological activity, which includes antioxidant properties, has attracted considerable attention [81]. Ellagic acid, a phenolic compound found in large amounts in dicotyledonous plants, has been shown in numerous studies to possess potent anti-inflammation and antioxidant properties. Furthermore, research shows that ellagic acid can lessen damage in neurodegenerative conditions such as AD, Parkinson’s disease, and cerebral ischemia by enhancing neuronal viability, reducing neuronal defects, and preventing neuronal damage [82]. A brand-new diabetes medication was made with plant flavonoids including epicatechin, catechin, and rutin, which have strong anti-inflammatory and antioxidant properties. Their combination can be improved through a mixture design experiment to produce a novel, safe, multitarget antidiabetic formulation, making it an effective combination for the management of diabetes and the associated complications. Rutin, catechin, and epicatechin all have strong antihyperglycemic properties; their synergistic combination assures a novel formulation that might actually be a viable alternative to current medications [83]. Accounting for roughly 59% of the total catechins, epigallocatechin gallate (EGCG) is the most prevalent flavanol. The beneficial effects of EGCG include its impact on metabolism, which lowers the risk of type 2 diabetes; its ability to block antimicrobial activity; and its antioxidant properties against neurodegenerative diseases such as AD [84]. In multi-infarct dementia model rats, nicotiflorin has protective effects such as energy metabolism failure, lowering memory dysfunction, and oxidative stress [85]. Astragalin has a wide spectrum of medicinal effects, including anti-inflammatory, antioxidant, neurological, cardioprotective, antidiabetic, and anticancer effects [86]. Resveratrol, quercetin, catechin, and gallic acid are examples of polyphenols that have antioxidant properties that prevent oxidative damage to DNA and inhibit LDL oxidation in vitro [87]. Antioxidant quinic acid has demonstrated anticancer activity by inducing apoptosis-mediated cytotoxicity in breast cancer cells. Additionally, it has shown a potent affinity for selectins, angiogenesis factors that are elevated in breast cancer tissue [88]. Tannic acid has antimutagenic and anticancer properties. Microorganisms can be killed by tannic acid (bacteria and viruses). Additionally, it functions as a homeostatic agent and an antioxidant. Tannic acid also has the ability to reduce the development of free radicals, which are responsible for a number of diseases, including those that affect the cardiovascular system, Parkinson’s disease, diabetes, and AD. Tannic acid also has demonstrated anticancer properties. Tannic acid is currently being researched as an organic polymer additive owing to its bioactive characteristics and its ability to improve the capabilities of materials for biomedical applications [89]. By restoring the normal expression levels of the genes related to insulin signaling and glucose metabolism that were disturbed in the liver of high-fat-diet-induced obese mice, hesperidin has the potential to have an antidiabetic effect [90].

An important indication of a compound’s potential antioxidant activity may be found in the reduction capacity of that substance. ROS and free radicals are capable of receiving electron donations from antioxidant compounds, which converts them into more stable and unreactive species [91]. The diversity, high amount of ingredients, and rich phenolic contents might contribute to the antioxidant potential of *P. granatum*. The reduction potentials of phenolic compounds in *P. granatum* were determined with reduction systems, including Cu^2+^, Fe^3+^, and Fe^3+^-TPTZ reducing abilities. The radical scavenging properties of *P. granatum* ethanol extracts was examined by DPPH and ABTS radical scavenging assays. *P. granatum* possesses reducing properties, which may neutralize oxidants and ROS.

The reduction of Fe^3+^(CN^−^)_6_ to Fe^2+^(CN^−^)_6_ and the absorbance resulting formation of Perl’s Prussian Blue complex after the addition of excess ferric ions (Fe^3+^) were used to measure the ability of *P. granatum* extracts to reduce Fe^3+^. The reducing power assay described by Oyaizu [44], with a minor modification, was applied to assess the reducing ability of *P. granatum* extracts [92]. In this assay, Fe^3+^ was converted to Fe^2+^ in the presence of reductants or plant extracts [93]. The addition of Fe^3+^ to compounds caused an Fe_4_[Fe(CN^−^)_6_]_3_ complex, with maximum absorption at 700 nm [94].

The chromogenic oxidant of neocuproine (Nc) was used in the CUPRAC method. Antioxidants reduce the cupric neocuproine complex [Cu(II)-Nc] to the cuprous neocuproine complex [Cu(I)-Nc], which exhibits maximum absorbance at 450 nm [95]. The CUPRAC method is a convenient, inexpensive, selective, stable of antioxidants [96,97]. The reducing capacity of pure compounds or plant extracts can be determined using the FRAP test. A ferric salt is utilized as an oxidant in the electron transfer process, which is the basis of the FRAP test. Due to its colored combination with TPTZ, which exhibits maximum absorbance at 593 nm, Fe^2+^ may be recorded spectrophotometrically [98]. The reducing capacity can be effectively ascertained using this method. First, in a redox-linked colorimetric reaction, the FRAP assay uses the sample’s antioxidants as reductants. Second, the FRAP assay procedure is fairly straightforward and is simple to standardize. The FRAP assay was created to assess the ability of biological fluids and aqueous solutions of pure compounds to reduce ferric ions. It has also been used to assess the antioxidant capacity of polyphenols [99]. In this study, we determined the Fe^3+^, Cu^2+^, and Fe^3+^-TPTZ reducing abilities of aqueous extract of *P. granatum* peel as concentration-dependent (10–30 μg/mL). In this test, the test solution’s color changed from yellow to various shades of green and blue depending on the antioxidant samples’ reducing power. A compound’s reducing capacity might be a good predictor of its potential antioxidant action.

In terms of the harm caused to living organisms by free radicals and ROS, radical scavenging is very important [100]. Due to its quick analysis time compared to other techniques, DPPH’s scavenging ability for free radicals has been commonly used to assess antioxidant activity [101]. For example, the DPPH^•^ test, which is based on scavenging of DPPH radicals to the non-radical form of DPPH-H, is commonly used to determine antioxidant activity [102,103]. A freshly made DPPH solution displays a deep purple hue with an absorption peak at 517 nm. When an antioxidant is present in the medium, this purple color typically vanishes. An indicator of the amount of free DPPH that has been reduced by the antioxidant is a decrease in absorbance [104]. As observed in this and previous studies, *P. granatum* has a comparable or better antioxidant potential relative to standard antioxidants. In another study, the IC_50_ values of acetone and ethanol extracts of *P. granatum* peel for DPPH scavenging activity were found to be 1.56 and 7.09 µg/mL, respectively [77]. The IC_50_ of methanolic extract of *P. granatum* for DPPH radical scavenging was reported to be 0.16 ± 0.07 mg/mL [105]. The IC_50_ values of aqueous and ethanolic extracts from *P. granatum* fruit peel for DPPH radical scavenging were found to be 471.7 and 509.16 g/mL, respectively, [106]. All analyses were performed in triplicate.

The ABTS radicals were produced in an ABTS/K_2_S_2_O_8_ system. The test is a decolorization approach in which the ABTS radical is created directly in a stable state prior to treatment with suspected antioxidants. The improved approach for producing ABTS^•+^ reported here involves the direct creation of a blue/green ABTS^•+^ chromophore via a reaction between ABTS and K_2_S_2_O_8_ [107]. One spectrophotometric technique used to assess the overall antioxidant ability of pure materials, mixtures, and beverages is based on the generation of an ABTS radical cation [108]. When compared to positive controls, the data clearly reveal that *P. granatum* approximated an effective ABTS^•+^ scavenging ability. *P. granatum* samples showed a radical scavenging effect higher than that of reference standard antioxidants. A lower IC_50_ value, as in DPPH free radical scavenging activity, suggests more ABTS^•+^ scavenging ability.

α-Glycosidase plays a crucial role in the metabolism of carbohydrates and is associated with diabetes, cancer, and viral infections. Because of its numerous biological functions, α-Glycosidase is regarded as a promising drug target [109]. Several α-glycosidase inhibitors have recently been found and are currently being researched. Acarbose and miglitol, two commonly prescribed diabetes medications, competitively inhibit α-glycosidase in the brush border of the small intestine. This prevents the hydrolysis of carbohydrates and reduces postprandial hyperglycemia [110]. α-Glycosidase inhibitors may play a significant role in the therapeutic approach to type 2 diabetes mellitus. Postprandial hyperglycemia is a notable and early defect in diabetic diseases, and lowering blood glucose levels can slow the progression of secondary complications related to diabetic diseases [111]. The results reveal that ethanol extract of *P. granatum* has less inhibitory effects than that of acarbose (IC_50_: 22,800 nM) [63]. According to various subsequent studies, IC_50_ value of *P. granatum* peel extract for inhibition of α-glycosidase activity was 5.56 2.23 µg/mL. Punicalagins may be responsible for this activity [112]. The ethanolic extract of *P. granatum* fruit peel demonstrated concentration-dependent inhibition of α-glucosidase, with activity ranging from 53.34 2.0 to 15.18 1.4 U/L. Aqueous extract, on the other hand, showed activity ranging from 65.48 1.8 to 20.2 1.3 U/L at the different tested concentrations [105]. The results of α-glycosidase inhibition of *P. granatum* extract are quite significant and indicate potential use of *P. granatum* for DM disease.

In order to properly digest carbohydrates, digestive enzymes such as α-amylase and α-glycosidase are essential glycoside hydrolases. Both of these enzymes are found on the cells that line the intestine, where they hydrolyze polysaccharides into monosaccharide units that can be absorbed. Certain inhibitors can block the actions of both digestive enzymes to reduce body weight and regulate blood glucose levels. A relatively safe source of inhibitors is plant-based food [113]. Because α-amylase plays a significant role in the digestion of dietary starches, its inhibition helps to prevent and control postprandial hyperglycemia. As a result, numerous studies have looked into and discovered the inhibition of α-amylase by natural products, such as plant extracts, in recent years [114]. *P. granatum* peel extracts in both aqueous and methanolic form were found to have no effect on the enzyme α-amylase in earlier research [115]. The acetone extract of *P. granatum* peel demonstrated excellent α-amylase inhibitory glycemic control potential, as well as dose-dependent but moderate antiglycation activity (IC_50_: 16.2 5.6 µg/mL), with 61% inhibition at 80 g/mL [77]. Measurements of the in vitro inhibition of α-glucosidase and α-amylase by *P. granatum* bark extracts were performed at two different concentrations (166 and 332 µg/mL) [115].

The most common and primary cause of dementia in the elderly is AD, a common neurodegenerative disease. The most significant biochemical change associated with AD is a decrease in AChE levels in the brain [116]. According to studies, the decline in acetyltransferase activity and choline (Ch) causes acetylcholine (ACh) to decrease as a neurotransmitter. As a result, cholinesterase (ChE) inhibitors have been the focus of research studies on the treatment of this illness as a symptomatic intervention [117]. AChE-inhibitory medicines are utilized in the treatment of AD. However, these medications have several undesired side effects. Therefore, research on use of novel AChE inhibitors with antioxidant ability is greatly needed [118]. It is known that the predominant AChE inhibitory effects are related to aromatic chemicals and, to a lesser extent, aliphatic molecules [119]. Although AChE inhibitors are used to treat AD, they can only bring about short-term relief. Medicinal herbs have long been known to rich in cholinesterase inhibitors. Phenolic chemicals are primarily responsible for medicinal plants’ suppression of cholinergic enzymes [120]. The in vitro cholinesterase-inhibitory effect of *P. granatum* peel extract is noteworthy, and its methanol extract was found to be more effective than its ethanol extract. The higher AChE activity of methanolic (IC_50_: 32 µg/mL) and ethanolic (IC_50_: 42 µg/mL) extract was correlated with the bioactive metabolite content of the extracts [121]. The inhibition level of *P. granatum* ethanol extract was slightly lower compared to that of tacrine.

Numerous diseases, including glaucoma, epilepsy, edema, and altitude sickness, are caused by the ubiquitous, physiologically dominant cytosolic isoform CA II [122]. CA isoform activation and inhibition are important therapeutic targets to treat a variety of diseases, including glaucoma, cancer, edema, obesity, epilepsy, hypertension, and osteoporosis [123]. CA II suppression reduces HCO_3_^−^ generation and, as a result, aqueous humor secretion, resulting in reduced ocular pressure [124]. Among them, glaucoma is a multifactorial optical disease that is mostly associated with high intraocular pressure (IOP), which can result in blindness. Therefore, hCA inhibitor medications such as acetazolamide, brinzolamide, and dorzolamide can reduce IOP after topical treatment [125].

One of the most prevalent Gram-positive bacteria that causes food poisoning among is *Staphylococcus aureus*, which is derived people who consumed contaminate food [126]. A Gram-negative bacterium called *Escherichia coli* is a part of the typical human flora. Preservatives are required to stop its growth because an enterohemorrhagic strain of *E. coli* has been implicated in severe cases of food poisoning [14]. Bacteria (*E. coli* and *S. aureus*) were resistant to all four antibiotics used as standards. Although some of them had a zone diameter of 10 mm, etc., they were considered resistant because they could not reach the standard sensitivity diameter according to the CLSI criteria.

Some of the extracts were resistant (R) because they did not form any zone diameter (N.D.). However, in some extracts, zones with 7–10 mm intervals, that is, areas in which the extract had an antimicrobial effect and the bacteria were destroyed, were observed. Discs with diameters of 7–10 mm formed at 50 μg (concentration-adjusted) ratios on each extraction disc are very good when compared to standard antibiotics, as observed in extracts that were completely zoneless, that is, resistant.

The benefits of *P. granatum* can be increased by drinking smoothies made from minor Mediterranean crop purées and *P. granatum* juice as a good way to increase the consumption of these healthy but underutilized fruits. The effects of an ethanol extract of *P. granatum* seeds on the central nervous system (CNS) in mice were studied. The results showed that *P. granatum* extract exhibit anxiolytic activity at all doses and induced increased sleeping latency and decreased sleeping time.

The effects of an ethanolic extract of *P. granatum* seeds on the CNS of mice were studied. The results showed that *P. granatum* extract exhibited anxiolytic activity at all dose levels and induced increased sleeping latency and decreased sleeping time [127]. The flavonoids in *P. granatum* vary greatly. For instance, flavonoids in plants can be found either in free form (aglycones) or linked to sugars. Glycosylated flavonoids are the most common, and glycosylated anthocyanidins, for example, are recognized as an essential flavonoid class known as anthocyanins. Anthocyanidins are light-sensitive and have been linked to sugars. *O*-glycosides are the most common type of flavonoid glycoside, but *C*-glycosides are also present. The benefits of P. granatum can be increased by drinking smoothies made from minor Mediterranean crop purées and *P. granatum* juice as a good way to increase consumption of these healthy but underutilized fruits [128,129].

## 5. Conclusions

Zivzik pomegranate (*Punica granatum*) has various qualities and contains quantities of bioactive secondary metabolites, phenolics, and flavonoids. This product, which is rich, nutritious, and contributes to human health, has been used since prehistoric times. In LC-MS/MS analysis, the major components detected in *P. granatum* extracts were ellagic acid, catechin, epigallocatechin gallate, epicatechin, nicotiflorin, astragalin, gallic acid, epigallocatechin, quinic acid, tannic acid, aconitic acid, hesperidin, isoquercitrin, rutin, fumaric acid, cosmosiin, luteolin, and epicatechin gallate. Furthermore, the *P. granatum* ethanol extract was found to be rich in phenolic contents, antioxidant ability, reducing power, AChE, α-glycosidase, α-amylase, and hCA II inhibition. *P. granatum* can also be used as a natural remedy to treat severe T2DM, AD, and glaucoma disease, as well as in food and pharmaceutical applications. From this perspective, inhibition studies on the AChE enzyme are planned to determine the anti-Alzheimer effects of WEZP and EEZP. In addition, the inhibition of CA II enzyme was analyzed to determine the link with glaucoma. Similarly, some studies have been carried out to identify the antidiabetic potential of *P. granatum* extracts on α-amylase and α-glycosidase. Additionally, Fe^2+^, Cu^2+^, and Fe^3+^-TPTZ reduction, as well as DPPH and ABTS scavenging, tests were performed to understand the antioxidant potential of *P. granatum*. Furthermore, total phenolic and flavonoid contents in *P. granatum* were established for both extracts. Finally, an analysis of the phenolic compounds was performed via LC-MS/MS to define the biological activity of the chemical profile of *P. granatum*. However, the possible cytotoxic or other undesirable effects of *P. granatum* should be more comprehensively detailed in the future.

## Figures and Tables

**Table 1:** Analytical method validation parameters and chemical profiles of WEZP and EEZP by LC-MS/MS analysis.

No	Analyte	RT *^a^*	M.I. (*m*/*z*) *^b^*	F.I. (*m*/*z*) *^c^*	Ion. Mode	Equation	*r^2 d^*	*RSD*% *^e^*		Linearity Range (mg/L)	*LOD*/*LOQ* (µg/L) *^f^*	Recovery (%)		*U ^g^*	Gr. No *^i^*	Phenolics	
								Interday	Intraday			Interday	Intraday			WEZP	EEZP
1	Quinic acid	3.0	190.8	93.0	Neg	*y* = −0.0129989 + 2.97989*x*	0.996	0.69	0.51	0.1–5	25.7/33.3	1.0011	1.0083	0.0372	1	44.662	17.460
2	Fumaric aid	3.9	115.2	40.9	Neg	*y* = −0.0817862 + 1.03467*x*	0.995	1.05	1.02	1–50	135.7/167.9	0.9963	1.0016	0.0091	1	N.D.	2.128
3	Aconitic acid	4.0	172.8	129.0	Neg	*y* = −0.7014530 + 32.9994*x*	0.971	2.07	0.93	0.1–5	16.4/31.4	0.9968	1.0068	0.0247	1	N.D.	8.190
4	Gallic acid	4.4	168.8	79.0	Neg	*y* = 0.0547697 + 20.8152*x*	0.999	1.60	0.81	0.1–5	13.2/17.0	1.0010	0.9947	0.0112	1	0.846	20.021
5	Epigallocatechin	6.7	304.8	219.0	Neg	*y* = −0.00494986 + 0.0483704*x*	0.998	1.22	0.73	1–50	237.5/265.9	0.9969	1.0040	0.0184	3	N.D.	19.148
6	Protocatechuic acid	6.8	152.8	108.0	Neg	*y* = 0.211373 + 12.8622*x*	0.957	1.43	0.76	0.1–5	21.9/38.6	0.9972	1.0055	0.0350	1	N.D.	0.371
7	Catechin	7.4	288.8	203.1	Neg	*y* = −0.00370053 + 0.431369*x*	0.999	2.14	1.08	0.2–10	55.0/78.0	1.0024	1.0045	0.0221	3	0.195	27.664
8	Gentisic acid	8.3	152.8	109.0	Neg	*y* = −0.0238983 + 12.1494*x*	0.997	1.81	1.22	0.1–5	18.5/28.2	0.9963	1.0077	0.0167	1	N.D.	N.D.
9	Chlorogenic acid	8.4	353.0	85.0	Neg	*y* = 0.289983 + 36.3926*x*	0.995	2.15	1.52	0.1–5	13.1/17.6	1.0000	1.0023	0.0213	1	N.D.	N.D.
10	Protocatechuic aldehyde	8.5	137.2	92.0	Neg	*y* = 0.257085 + 25.4657*x*	0.996	2.08	0.57	0.1–5	15.4/22.2	1.0002	0.9988	0.0396	1	N.D.	0.154
11	Tannic acid	9.2	182.8	78.0	Neg	*y* = 0.0126307 + 26.9263*x*	0.999	2.40	1.16	0.05–2.5	15.3/22.7	0.9970	0.9950	0.0190	1	1.694	12.287
12	Epigallocatechin gallate	9.4	457.0	305.1	Neg	*y* = −0.0380744 + 1.61233*x*	0.999	1.30	0.63	0.2–10	61.0/86.0	0.9981	1.0079	0.0147	3	0.090	25.600
13	1,5-Dicaffeoylquinic acid	9.8	515.0	191.0	Neg	*y* = −0.0164044 + 16.6535*x*	0.999	2.42	1.48	0.1–5	5.8/9.4	0.9983	0.9997	0.0306	1	N.D.	N.D.
14	4-OH Benzoic acid	10.5	137.2	65.0	Neg	*y* = −0.0240747 + 5.06492*x*	0.999	1.24	0.97	0.2–10	68.4/88.1	1.0032	1.0068	0.0237	1	N.D.	N.D.
15	Epicatechin	11.6	289.0	203.0	Neg	*y* = −0.0172078 + 0.0833424*x*	0.996	1.47	0.62	1–50	139.6/161.6	1.0013	1.0012	0.0221	3	N.D.	24.210
16	Vanillic acid	11.8	166.8	108.0	Neg	*y* = −0.0480183 + 0.779564*x*	0.999	1.92	0.76	1–50	141.9/164.9	1.0022	0.9998	0.0145	1	N.D.	N.D.
17	Caffeic acid	12.1	179.0	134.0	Neg	*y* = 0.120319 + 95.4610*x*	0.999	1.11	1.25	0.05–2.5	7.7/9.5	1.0015	1.0042	0.0152	1	N.D.	0.096
18	Syringic acid	12.6	196.8	166.9	Neg	*y* = −0.0458599 + 0.663948*x*	0.998	1.18	1.09	1–50	82.3/104.5	1.0006	1.0072	0.0129	1	N.D.	N.D.
19	Vanillin	13.9	153.1	125.0	Poz	*y* = 0.00185898 + 20.7382*x*	0.996	1.10	0.85	0.1–5	24.5/30.4	1.0009	0.9967	0.0122	1	N.D.	0.201
20	Syringic aldehyde	14.6	181.0	151.1	Neg	*y* = −0.0128684 + 7.90153*x*	0.999	2.51	0.77	0.4–20	19.7/28.0	1.0001	0.9964	0.0215	1	N.D.	N.D.
21	Daidzin	15.2	417.1	199.0	Poz	*y* = 9.45747 + 152.338*x*	0.996	2.25	1.32	0.05–2.5	7.0/9.5	0.9955	1.0017	0.0202	2	N.D.	N.D.
22	Epicatechin gallate	15.5	441.0	289.0	Neg	*y* = −0.0142216 + 1.06768*x*	0.997	1.63	1.28	0.1–5	19.5/28.5	0.9984	0.9946	0.0229	3	N.D.	1.060
23	Piceid	17.2	391.0	135/106.9	Poz	*y* = 0.00772525 + 25.4181*x*	0.999	1.94	1.16	0.05–2.5	13.8/17.8	1.0042	0.9979	0.0199	1	N.D.	0.23
24	*p*-Coumaric acid	17.8	163.0	93.0	Neg	*y* = 0.0249034 + 18.5180*x*	0.999	1.92	1.43	0.1–5	25.9/34.9	1.0049	1.0001	0.0194	1	N.D.	0.874
25	Ferulic acid-D3-IS *^h^*	18.8	196.2	152.1	Neg	N.A.	N.A.	N.A.	N.A.	N.A.	N.A.	N.A.	N.A.	0.0170	1	N.A.	N.A.
26	Ferulic acid	18.8	192.8	149.0	Neg	*y* = −0.0735254 + 1.34476*x*	0.999	1.44	0.53	1–50	11.8/15.6	0.9951	0.9976	0.0181	1	N.D.	N.D.
27	Sinapic acid	18.9	222.8	193.0	Neg	*y* = −0.0929932 + 0.836324*x*	0.999	1.45	0.52	0.2–10	65.2/82.3	1.0031	1.0037	0.0317	1	N.D.	N.D.
28	Coumarin	20.9	146.9	103.1	Poz	*y* = 0.0633397 + 136.508*x*	0.999	2.11	1.54	0.05–2.5	214.2/247.3	0.9950	0.9958	0.0383	1	N.D.	N.D.
29	Salicylic acid	21.8	137.2	65.0	Neg	*y* = 0.239287 + 153.659*x*	0.999	1.48	1.18	0.05–2.5	6.0/8.3	0.9950	0.9998	0.0158	1	N.D.	N.D.
30	Cynaroside	23.7	447.0	284.0	Neg	*y* = 0.280246 + 6.13360*x*	0.997	1.56	1.12	0.05–2.5	12.1/16.0	1.0072	1.0002	0.0366	2	N.D.	0.926
31	Miquelianin	24.1	477.0	150.9	Neg	*y* = −0.00991585 + 5.50334*x*	0.999	1.31	0.95	0.1–5	10.6/14.7	0.9934	0.9965	0.0220	2	N.D.	N.D.
32	Rutin-D3-IS *^h^*	25.5	612.2	304.1	Neg	N.A.	N.A.	N.A.	N.A.	N.A.	N.A.	N.A.	N.A.	N.A.	2	N.A.	N.A.
33	Rutin	25.6	608.9	301.0	Neg	*y* = −0.0771907 + 2.89868*x*	0.999	1.38	1.09	0.1–5	15.7/22.7	0.9977	1.0033	0.0247	2	0.024	2.732
34	Isoquercitrin	25.6	463.0	271.0	Neg	*y* = −0.111120 + 4.10546*x*	0.998	2.13	0.78	0.1–5	8.7/13.5	1.0057	0.9963	0.0220	2	0.038	4.056
35	Hesperidin	25.8	611.2	449.0	Poz	*y* = 0.139055 + 13.2785*x*	0.999	1.84	1.35	0.1–5	19.0/26.0	0.9967	1.0043	0.0335	2	0.063	6.136
36	*o*-Coumaric acid	26.1	162.8	93.0	Neg	*y* = 0.00837193 + 11.2147*x*	0.999	2.11	1.46	0.1–5	31.8/40.4	1.0044	0.9986	0.0147	1	N.D.	N.D.
37	Genistin	26.3	431.0	239.0	Neg	*y* = 1.65808 + 7.57459*x*	0.991	2.01	1.28	0.1–5	14.9/21.7	1.0062	1.0047	0.0083	2	N.D.	N.D.
38	Rosmarinic acid	26.6	359.0	197.0	Neg	*y* = −0.0117238 + 8.04377*x*	0.999	1.24	0.86	0.1–5	16.2/21.2	1.0056	1.0002	0.0130	1	N.D.	N.D.
39	Ellagic acid	27.6	301.0	284.0	Neg	*y* = 0.00877034 + 0.663741*x*	0.999	1.57	1.23	0.4–20	56.9/71.0	1.0005	1.0048	0.0364	1	1.518	199.967
40	Cosmosiin	28.2	431.0	269.0	Neg	*y* = −0.708662 + 8.62498*x*	0.998	1.65	1.30	0.1–5	6.3/9.2	0.9940	0.9973	0.0083	2	0.019	2.036
41	Quercitrin	29.8	447.0	301.0	Neg	*y* = −0.00153274 + 3.20368*x*	0.999	2.24	1.16	0.1–5	4.8/6.4	0.9960	0.9978	0.0268	2	N.D.	N.D.
42	Astragalin	30.4	447.0	255.0	Neg	*y* = 0.00825333 + 3.51189*x*	0.999	2.08	1.72	0.1–5	6.6/8.2	0.9968	0.9957	0.0114	2	0.243	20.551
43	Nicotiflorin	30.6	592.9	255.0/284.0	Neg	*y* = 0.00499333 + 2.62351*x*	0.999	1.48	1.23	0.05–2.5	11.9/16.7	0.9954	1.0044	0.0108	2	0.273	23.535
44	Fisetin	30.6	285.0	163.0	Neg	*y* = 0.0365705 + 8.09472*x*	0.999	1.75	1.19	0.1–5	10.1/12.7	0.9980	1.0042	0.0231	3	N.D.	N.D.
45	Daidzein	34.0	253.0	223.0	Neg	*y* = −0.0329252 + 6.23004*x*	0.999	2.18	1.73	0.1–5	9.8/11.6	0.9926	0.9963	0.0370	3	N.D.	N.D.
46	Quercetin-D3-IS *^h^*	35.6	304.0	275.9	Neg	N.A.	N.A.	N.A.	N.A.	N.A.	N.A.	N.A.	N.A.	N.A.	3	N.A.	N.A.
47	Quercetin	35.7	301.0	272.9	Neg	*y* = +0.00597342 + 3.39417*x*	0.999	1.89	1.38	0.1–5	15.5/19.0	0.9967	0.9971	0.0175	3	N.D.	0.136
48	Naringenin	35.9	270.9	119.0	Neg	*y* = −0.00393403 + 14.6424*x*	0.999	2.34	1.69	0.1–5	2.6/3.9	1.0062	1.0020	0.0392	3	0.004	0.234
49	Hesperetin	36.7	301.0	136.0/286.0	Neg	*y* = +0.0442350 + 6.07160*x*	0.999	2.47	2.13	0.1–5	7.1/9.1	0.9998	0.9963	0.0321	3	N.D.	N.D.
50	Luteolin	36.7	284.8	151.0/175.0	Neg	*y* = −0.0541723 + 30.7422*x*	0.999	1.67	1.28	0.05–2.5	2.6/4.1	0.9952	1.0029	0.0313	3	0.005	1.126
51	Genistein	36.9	269.0	135.0	Neg	*y* = −0.00507501 + 12.1933*x*	0.999	1.48	1.19	0.05–2.5	3.7/5.3	1.0069	1.0012	0.0337	3	N.D.	N.D.
52	Kaempferol	37.9	285.0	239.0	Neg	*y* = −0.00459557 + 3.13754*x*	0.999	1.49	1.26	0.05–2.5	10.2/15.4	0.9992	0.9990	0.0212	3	N.D.	0.357
53	Apigenin	38.2	268.8	151.0/149.0	Neg	*y* = 0.119018 + 34.8730*x*	0.998	1.17	0.96	0.05–2.5	1.3/2.0	0.9985	1.0003	0.0178	3	N.D.	0.338
54	Amentoflavone	39.7	537.0	417.0	Neg	*y* = 0.727280 + 33.3658*x*	0.992	1.35	1.12	0.05–2.5	2.8/5.1	0.9991	1.0044	0.0340	3	0.013	0.009
55	Chrysin	40.5	252.8	145.0/119.0	Neg	*y* = −0.0777300 + 18.8873*x*	0.999	1.46	1.21	0.05–2.5	1.5/2.8	0.9922	1.0050	0.0323	3	N.D.	N.D.
56	Acacetin	40.7	283.0	239.0	Neg	*y* = −0.559818 + 163.062*x*	0.997	1.67	1.28	0.02–1	1.5/2.5	0.9949	1.0011	0.0363	3	N.D.	N.D.

*^a^* RT: retention time; *^b^* MI (*m/z)*: molecular ions of the standard analytes (*m*/*z* ratio); *^c^* FI (*m/z)*: fragment ions; *^d^ r*^2^: coefficient of determination; *^e^ RSD*: relative standard deviation; *^f^ LOD*/*LOQ* (µg/L): limit of detection/quantification; *^g^ U* (%): percent relative uncertainty at 95% confidence level (*k* = 2); *^h^* IS: internal standard; *^i^* Gr. No: grouping of internal standards (these numbers indicate which IS stands for which phenolic compound); N.D.: not detected; N.A.: not applicable.

**Table 2:** Fe^3+^, Cu^2+^, and Fe^3+^-TPTZ reducing capabilities of *P. granatum* extracts, juice, and positive controls at 30 μg/mL (BHA: butylated hydroxyanisole; BHT: butylated hydroxytoluene).

Antioxidant	Fe^3+^ Reducing *		Cu^2+^ Reducing *		Fe^3+^-TPTZ Reducing *	
	λ_700_	r^2^	λ_450_	r^2^	λ_593_	r^2^
BHA	2.319 ± 0.041 ^a^	0.9629	2.849 ± 0.020 ^a^	0.9994	2.151 ± 0.020 ^b^	0.9367
BHT	1.873 ± 0.152 ^b^	0.9918	2.865 ± 0.038 ^a^	0.9991	2.031 ± 0.190 ^b^	0.9670
Trolox	2.334 ± 0.167 ^a^	0.9997	2.555 ± 0.022 ^a^	0.9987	2.108 ± 0.026 ^b^	0.9291
α-Tocopherol	2.778 ± 0.248 ^a^	0.9999	2.185 ± 0.110 ^b^	0.9986	2.434 ± 0.103 ^a^	0.8714
WEZP peel	1.278 ± 0.143 ^c^	0.9995	0.927 ± 0.022 ^c^	0.9965	1.903 ± 0.052 ^b^	0.9875
WEZP seed	0.229 ± 0.033 ^d^	0.9252	0.114 ± 0.034 ^d^	0.8485	0.483 ± 0.023 ^c^	0.9124
EEZP peel	1.219 ± 0.028 ^c^	0.9253	0.878 ± 0.017 ^c^	0.9967	2.086 ± 0.080 ^b^	0.9866
EEZP seed	0.258 ± 0.005 ^d^	0.9712	0.194 ± 0.008 ^d^	0.9974	0.606 ± 0.011 ^c^	0.9471
*P. granatum* juice	1.810 ± 0.149 ^b^	0.4020	2.790 ± 0.045 ^a^	0.9999	2.230 ± 0.010 ^b^	0.9056

* All values are averages of three parallel measurements (n = 3) and presented as mean ± SD. Different letters in the same column indicate a significant difference between the means (*p* < 0.05 regarded as significant).

**Table 3:** IC_50_ values (μg/mL) of DPPH^•^ and ABTS^•+^ scavenging activities of *P. granatum* and standards.

Antioxidant	DPPH^•^ Scavenging		ABTS^•+^ Scavenging	
	IC_50_	r^2^	IC_50_	r^2^
BHA	6.86	0.9949	6.35	0.9746
BHT	49.50	0.9957	12.60	0.9995
Trolox	6.03	0.9925	16.50	0.9775
α-Tocopherol	7.70	0.9961	18.72	0.9347
Ascorbic acid	5.82	0.9668	11.74	0.9983
WEZP peel	31.50	0.9995	8.80	0.9178
WEZP seed	-	-	-	-
EEZP peel	16.10	0.9310	5.90	0.9669
EEZP seed	-	-	-	-
*P. granatum* Juice	-	-	-	-

**Table 4:** The half-maximal inhibition concentration (IC_50_; µg/mL) of *P. granatum* towards acetylcholinesterase, α-glycosidase, α-amylase, and carbonic anhydrase II enzymes.

Enzyme	AChE		hCA II		α-Glycosidase		α-Amylase	
	IC_50_	r^2^	IC_50_	r^2^	IC_50_	r^2^	IC_50_	r^2^
WEZP peel	20.0	0.9976	36.4	0.9957	28.8	0.9420	494.3	0.7705
WEZP seed	20.4	0.9851	144.5	0.9906	6.4	0.8819	375.8	0.8193
EEZP peel	19.7	0.9869	106.3	0.9941	7.3	0.9399	317.7	0.7778
EEZP seed	17.8	0.9976	30.4	0.8881	-	-		
*P. granatum* juice	22.6	0.9951	94.0	0.9909	27.1	0.9665	70.1	0.9999
Standards	5.97 ^1^	0.9706	8.4 ^2^	0.9825	22,800 ^3^	-		

^1^ Acetazolamide (AZA) was used as a standard inhibitor for carbonic anhydrase II isoenzyme. ^2^ Tacrine was used as a standard inhibitor for acetylcholinesterase. ^3^ Acarbose was used as a standard inhibitor for α-glycosidase and α-amylase enzymes [63].

**Table 5:** Antimicrobial activities of *P. granatum* extracts (50 µg/disk). Amc 30: amoxycillin/clavulanic acid antimicrobial susceptibility disks (30 µg/disk); Sxt 25: trimethoprim/sulfamethoxazole (25 µg/disk); Cip 5: ciprofloxacin (5 µg/disk); Gnt 10: gentamicin (10 µg/disk).

Sample	Antimicrobial Zone (mm)	
	*Escherichia coli* ATCC 39628	*Staphylococcuc aureus* ATCC 25923
WEZP peel	8	8
WEZP seed	R, N.D.	9
EEZP peel	R, N.D.	R, N.D.
EEZP seed	R, N.D.	R, N.D.
*P. granatum* juice	10	R, N.D.
Amc/Clav-30	10, R	R, N.D.
Sxt-25	R, N.D.	R, N.D.
Cip-5	R, N.D.	10, R
Gnt-10	11, R	R, N.D.

N.D.: activity not detected at this concentration; S: sensitivity; R: resistant.

## Data Availability

Data are publicly available in an accessible repository.
